# Perception of facial trustworthiness in mild cognitive impairment

**DOI:** 10.1177/13872877261426496

**Published:** 2026-03-13

**Authors:** Marta Granadeiro, Leonel Garcia-Marques, Marco Torrado, Isabel Pavão Martins

**Affiliations:** 1 Laboratório de Estudos de Linguagem, Centro de Estudo Egas Moniz, Faculty of Medicine, University of Lisbon, Lisbon, Portugal; 2 Gulbenkian Institute for Molecular Medicine, Lisbon, Portugal; 3 CUF Academic Center, Lisbon, Portugal; 4 Faculdade de Psicologia, CICPSI, 37809 Universidade de Lisboa, Lisbon, Portugal; 5 Headache Outpatient Clinic, Department of Neurosciences, ULS Santa Maria, Lisbon, Portugal

**Keywords:** Alzheimer's disease, behavioral impairment, mild cognitive impairment, social cognition, social trait inference, trustworthiness

## Abstract

**Background:**

Impaired social behavior in mild cognitive impairment (MCI) represents an important burden for caregivers and its presence is associated with increased risk of conversion to dementia. Social cognition provides a relevant framework for investigating functional and social outcomes of these patients but is still based on a very narrow number of domains and lacks robust connection to clinical outcomes.

**Objective:**

The present study aims to investigate social trait inference, our ability to form judgements about others, in MCI patients and how it relates to functional outcomes.

**Methods:**

We used Signal Detection Theory's measures of sensitivity and bias in perceiving facial trustworthiness.

**Results:**

We found that increasing education level decreased the difference in sensitivity between MCI patients and controls. Importantly, we found that higher impairment in activities of daily living was associated with perceiving others as less trustworthy. In controls, lower cognitive integrity was also related to perceiving others as less trustworthy.

**Conclusions:**

Our results have important implications for the understanding of changes in social perception in MCI. Education may have a protective role in delaying the onset of impairment in social trait inference. Also, we show evidence that the age-associated positivity bias depends on higher cognitive integrity. Our results have implications for patient care and provide additional characterization of social perception in these patients.

## Introduction

Dementia is a long-known debilitating disease.^1^ Most dementias are preceded by an early stage of cognitive decline and behavioral changes. Mild cognitive impairment (MCI) has been one of the most widely used terms to describe those with subtle cognitive decline from previous functioning with a higher probability of developing dementia.^2^ Most criteria for MCI include observable cognitive decline, without or with very mild impairment in functionality and behavioral changes. More recently, however, the presence of social cognitive impairment in these patients has gained increased attention, especially in relation to interpersonal difficulties, behavioral impairment and neuropsychiatric symptoms in these patients.^3^

Social cognitive frameworks comprise the study of human perception, interaction and decision-making based on social information. The two most widely studied domains of social cognition in neuroscience have been emotion recognition and theory of mind. Emotion recognition focuses on how we perceive emotional expressions.^4^ Theory of mind is the ability to attribute mental states, intentions and motivations.^5^ Meta-analysis results show a moderate effect size of the impairment in emotion recognition and theory of mind in MCI patients.^3,6^ Additionally, when comparing MCI patients with dementia patients, the latter perform significantly worse,^7^ meaning that social cognitive function follows the same downward pathway of other cognitive functions in dementia. However, three limitations have characterized studies based on these two domains. The first is the absence of a clear relationship between emotion recognition or theory of mind with social functioning and neuropsychiatric symptoms.^6^ Secondly, characterizing social cognitive function with just two domains gives a very narrow understanding of these functions. Finally, the importance of these impairments in the treatment and other clinical outcomes of patients is not well understood.^8^ To address and better characterize social cognitive impairment and social functioning in MCI patients, a broader study of social cognition is necessary. In this study we focus on social trait inference, another social cognitive domain.

Social trait inference is one of the most extensively investigated domains of social cognition in the healthy population. It focuses on how we form judgements of others, by inferring social traits, even from the scarcest of information.^9,10^ These inferences have proven important for relevant social outcomes. In court decisions, the higher the ratings of baby-facedness^11,12^ of the defendant the lower the probability of the court deciding against the defendant.^13^ Perceived competence of faces predicts voting preferences and decisions.^14,15^ Importantly, the influence of these impressions persists even after educating people about their over reliance on facial features and after prioritizing relevant factual information.^16^ Understanding how MCI patients perceive others is of great importance. In patient-doctor relationship, patient perception of physicians is related to increased satisfaction and adherence, decreased anxiety, better diagnostic and clinical outcomes and patient enablement.^17^ Also, patients with cognitive impairment are at higher risk of being exploited by others,^18^ which may be related to difficulties perceiving them.

The neural correlates of social trait inference^19^ overlap with regions affected in the majority of neurodegenerative diseases, including Alzheimer's disease (AD) and cerebrovascular disease, which are the two most common causes of MCI. Those authors found that negative evaluation of face stimuli was associated with increased activity in the right amygdala, while positive evaluation of face stimuli was associated with increased activity of the caudate, nucleus accumbens, medial orbitofrontal cortex, thalamus, ventromedial prefrontal cortex and cingulate cortex.^19^ Also, the amygdala has been signaled as a site of early deposition of neurofibrillary tangles in histological postmortem studies and imaging studies of AD patients.^20^ In subcortical vascular cognitive impairment,^21^ resting-state MRI studies have identified changes in functional connectivity in areas such as the cingulate cortex, medial prefrontal cortex, thalamus and orbitofrontal cortex that are thought to also play a role in social trait inference.^22^

In the present study we focus on the perception of trustworthiness, one of the main and most important traits in person perception.^23^ Trustworthiness has been described as the perception of others’ intentions^24–26^ and it is crucial for approach/avoidance behavior.^27^ It is also considered to be a hallmark of human cooperation^28,29^ and our sensitivity to trustworthiness is present as early as 7 months old.^23^ Lastly, the perception of trustworthiness is intimately rooted in the perceived valence of emotional expressions, as we tend to infer trustworthiness based on the resemblance to positive or negative emotional expressions.^23,30^ As an emotion recognition impairment has already been described in MCI patients, and because trustworthiness perception is linked to emotion perception,^23,30^ it could be expected for trustworthiness perception to be also impaired. We used a two-alternative forced-choice task to investigate the ability to discriminate consensually rated facial trustworthiness in patients with MCI. Signal Detection Theory^31^ allows us to understand if there are differences in the sensitivity to facial trustworthiness between MCI patients and healthy controls. Following previous literature on emotion recognition, we expect lower sensitivity to facial trustworthiness in patients with MCI compared to healthy controls.

Signal Detection Theory also measures response bias, the tendency to answer in a more negative or positive way. Response bias could also inform about a general tendency for MCI patients to be more suspicious or more trustful concerning other people. Usually, higher response bias are called conservative bias (considering other people untrustworthy) and lower response bias are called liberal bias (considering other people trustworthy). A positivity effect has been described in healthy aging, which corresponds to the systematic observation that healthy older adults, in comparison to young adults, show a preference for the processing of positive material in memory, attention and perception tasks.^32^ One hypothesis, the socioemotional selective theory (SST; see^33^), predicts that this positivity effect depends on cognitive control processes and it is mainly observed in the healthy brain. The aging-brain model,^34^ on the other hand, predicts that the positivity bias derives from a decline in cognitive function and is higher for those with cognitive impairment. A more conservative bias in the MCI group, in comparison to the healthy controls, would support SST, while a more liberal bias in the MCI group would support the aging-brain model.

In this study we address two additional questions. On one hand, some authors argue that social cognition impairment in these patients is secondary to a more general decline in cognition.^3^ Others, however, have shown that performance on social cognitive tasks does not correlate with performance on other ‘standard’ cognitive tasks and, thus, that social cognition is functionally independent.^35^ Our study addressed the role of other cognitive domains on social trait inference in order to determine its relative independence. Another important matter concerns the role of demographic variables, namely if variables like age, education and sex influence performance on social cognitive tasks, as they do on ‘standard’ cognitive tasks. Like emotion recognition, the inference of social traits has been found to have a very similar structure across cultures,^26,36^ it is present relatively early in development^23^ and is thought to have an evolutionary basis.^12^ This would seem to imply that these functions would be robust to early cognitive decline and would not vary as a function of demographic variables. Nevertheless, studies on emotion recognition have systematically shown that this function is not intact in MCI and if we consider other cognitive functions, educational level is protective of its decline.^37^ A possible protective role of demographic variables on decline in social cognitive function might inform not only about which patients might be at greater risk of experiencing early social cognitive impairment, but also, which patients might already have a social cognitive impairment that would not yet be observable in neuropsychological testing.

To our knowledge, this is the first study investigating social trait inference in MCI patients. Its novelty could contribute to a more comprehensive understanding of impairments on social cognition in these patients by 1) broadening the domains of study of social cognition, providing a better characterization of cognitive and functional outcomes, 2) providing additional evidence for the relative functional independence of social cognition, 3) signaling possible protecting factors in demographic variables.

## Methods

### Study design and participants

We performed a prospective, observational, case-control study that included MCI patients. Patients were referred from a neurology outpatient clinic to cognitive assessment due to cognitive complaints. After the assessment, they were asked to participate in the study. Informed consent was mandatory to proceed to study participation, and it was emphasized that study participation was voluntary and was not necessary for other clinical purposes. The study protocol and informed consent were in accordance with Helsinki Declaration and were approved by the ethics committee of both the Lisbon Academic Medical Centre and CUF Academic Centre.

Sixty-six patients who fulfilled criteria for MCI^38^ were recruited for the study. Inclusion criteria were 1) complaints of cognitive decline (self/informant report of decline over time), 2) cognitive impairment, 3) preserved functionality. Because of known cultural and ethnic differences in the perception of social traits^26^ we only included Caucasian participants. Exclusion criteria were: 1) previous neurologic or psychiatric disorder, 2) decreased visual acuity that precluded from performing tasks with visual stimuli, 3) fulfilling criteria for dementia.^39^ Two patients were excluded because they did not evidence objective cognitive impairment, one patient asked to stop, and five patients fulfilled criteria for dementia. The final MCI patient sample was fifty-eight.

Sixty-two healthy participants were recruited as controls from a day center in the city of Lisbon and from family members that accompanied patients in the hospital. Inclusion criteria was 1) scoring within Portuguese normative data on the Montreal Cognitive Assessment (MoCA)^40^ and 2) being Caucasian. Exclusion criteria was 1) previous history of neurological or psychiatric disorder, 2) subjective cognitive complaints, and 3) decreased visual acuity that precluded from performing the tasks. Healthy controls from the day center were assessed at the day center in order to avoid additional expenses and interference with daily activities. The remaining controls were assessed at the same place as the MCI patients. Two controls were excluded due to previous neurological disease (stroke and epilepsy), one was excluded due to subjective cognitive complaints, two were excluded due to answering all “yes” or “no” in the social trait inference task, one asked to stop and eight were excluded due to scoring lower than one standard-deviation from normative data in the MoCA. The final sample included forty-eight healthy controls.

Demographic information about both patients and controls is shown in Table 1.

**Table 1.** Demographic information (age, education, sex, and handedness) and MoCA scores.

	MCI (n = 58)	HC (n = 48)	Total (n = 105)	p
Age (y)				
Mean (sd)	74.28 (9.35)	70.62 (9.41)	72.62 (9.51)	0.05^a^
range	48–87	48–90	48–90	
Education (y)				
Mean (sd)	8.48 (4.92)	10.29 (5.3)	9.3 (5.15)	0.07^a^
range	2–17	0–21	0–21	
Sex (%)				
Female	27 (46.6%)	34 (70.8%)		0.02^b^
Handedness (%)				
Right-handed	57 (98.3%)	42 (87.5%)		
Left-handed	1 (1.7%)	5 (10.4%)		
Ambidextrous	0 (0%)	1 (2.1%)		
MoCA (sd)	-	24.55 (2.8)		
MoCA Adjusted (sd)	-	0.15 (0.72)		

MoCA: Montreal Cognitive Assessment. MoCA Adjusted is MoCA score corrected for age and education (z-score).

^a^ one-way analysis of variance.

^b^ chi-squared test.

### Stimuli

40 photographs of faces in color from the Chicago Face Database were used as stimuli. Faces were selected based on published normative data of perceived trustworthiness,^41^ where the 20 face stimuli rated highest in perceived trustworthiness (M = 4.33, SD = 0.17) and the 20 face stimuli rated lowest in perceived untrustworthy (M = 2.52, SD = 0.16) were chosen. Stimuli were balanced for sex. It is important to note that when referring to “trustworthy” and “untrustworthy” faces we mean faces that were consensually rated as trustworthy or untrustworthy, respectively, by a normative sample. Also, we measured perceived trustworthiness for which actual objective trustworthiness is not relevant.

### Procedure

*Neuropsychological assessment.* Patients were subjected to a formal neuropsychological assessment, were given an informed consent, performed the social trait inference task and afterwards we performed a semi-structured interview with an informant caregiver for the filling of the Blessed Dementia Scale. The neuropsychological assessment took around 1.5 to 2 h. Diagnosis of MCI was determined using consensual established criteria.^38^ Functional impairment was measured through the total score of the Blessed Dementia Scale^42^ wherein total scores higher than 4 involved patient exclusion, as this is the cut-off point for dementia. The cognitive battery included three tests of each cognitive domain, namely, memory, attention, executive function and visuospatial/visuoperceptive ability. The tests used and mean scores are described in Table 2 (and in Supplemental Table 2 and Supplemental Procedure).

**Table 2.** Mean scores and standard deviation of Dprime, criterion, attention composite, memory composite, executive composite, visual composite, depressive symptoms and functionality.

		MCI		HC	
		M	SD	M	SD
*Dprime*		1.08	0.66	1.32	0.62
criterion		−0.05	0.56	−0.07	0.47
Attention		−0.08	1.06	-	
Memory		−0.87	1.15	-	
Executive		−0.62	0.68	-	
Visual		−1.41	3.64	-	
GDS		5.04	3.65	-	
BDS	ADL	0.83	0.74	-	
	Behavioural change	1.20	1.16	-	
	Total	2.03	1.24	-	

GDS-15: Geriatric Depression Scale; ADL: Activities of Daily Living; BDS: Blessed Dementia Scale.

*Social trait inference task.* This task was programmed in the Opensesame software.^43^ In each trial, a fixation signal in the center of a white background was presented in a computer screen for 500 ms. This was followed by a colored photograph of a neutral face (i.e., without emotional expression). The participants saw each face, individually, in each trial and the face was displayed until a response was given (illustration of the task in Supplemental Figure 1). Instructions were that the participants would see unfamiliar faces and had to decide if the person was “trustworthy” (pressing number 1) or not (pressing number 9), meaning if the person was someone they thought they could trust. It was also pointed that they did not have a time limit to answer but that they should decide based on their first impression. When in doubt, participants were prompted to choose the one they were more inclined to, even if not certain. The question “Do you think this person is trustworthy?” stayed in the screen until a response was given.

As the majority of participants were not regular keyboard users and to avoid fatigue effects the patient responded orally and the researcher (in the back of the screen) pressed the corresponding buttons. The researcher could not see any of the stimuli being presented.

Stimuli were presented in random order, each participant saw all the faces once and total task duration was 10 min.

### Statistical analysis

All statistical analysis were performed using R software.^44^

Differences in demographic variables between MCI patients and controls were tested for age, education and sex. We used a one-way analysis of variance (ANOVA) for age and education and chi-squared test for sex distribution.

Signal Detection Theory is a framework from psychology that is used to investigate discrimination ability for the presence or absence of a signal.^31^ For our study, it provides two measures of performance. The first measure is *Dprime* (or *d*’) which is a measure of sensitivity or discrimination ability, meaning how well a person can discriminate whether the face is trustworthy or not. The second measure is *C* (or criterion), a measure of response bias, that indicates the threshold for each person to answer ‘yes’ or ‘no’. If the criterion is high (*C* > 0), then the probability that the person considers the face trustworthy is low. If, on the other hand, the criterion is low (*C* < 0), the probability that the person considers the face trustworthy is higher.

*Dprime* and *C* values for each participant were computed through function ‘*Dprime*’ of the ‘psycho’ package.^45^ This function computes *Dprime* values based on the difference between z-value of the hit rate (z(Hits)) and z-value of the false alarm rate ((z(False Alarms). It also computes *C* values based on the formula −0,5(z(False Alarms) + z(Hits)).^46^ In this study we defined hit as answering ‘yes’ to a trustworthy face and false alarm as answering ‘yes’ to an untrustworthy face.

Statistical analysis for the group differences in *Dprime* scores was based on multiple linear regression and were performed using function ‘lm’ from package ‘stats’.^44^ In the model, we included group, education, age and sex of participants as predictor variables. Group differences in criterion were based on a similar model, including the same predictor variables. Interaction terms were included if they improved model fitness as determined by *F*-test. The presence of influential data points in the regression models was determined by cook's D. Cook's D was computed using function ‘check_outliers’ from package ‘performance’.^47^

To test if the results were based on trustworthiness perception and not in the perception of other social traits we also tested the same model for dominance and attractiveness. The same forty faces were classified as high or low in dominance and as high or low in attractiveness based on the same normative data used for trustworthiness classification. Faces with mean ratings lower than the general mean for that trait were considered low on that trait and faces with mean ratings higher than the general mean for that trait were considered high on that trait (more detailed information in Supplemental Table 3). A *Dprime* and criterion were also computed for dominance and attractiveness based on the same responses (as for trustworthiness). Two separate models were tested using multiple linear regression with group, education, age and sex as predictor variables. One model included dominance *Dprime* as the dependent variable and the other included attractiveness *Dprime* as dependent variable.

We performed pairwise Pearson correlations between *Dprime* and criterion scores and other cognitive and functional measures in MCI patients using the function ‘rcorr’ from the ‘Hmisc’ package.^48^ For cognitive measures, we computed a composite for each of the four cognitive domains assessed (memory, executive function, attention and visuoperception) through the mean of z-scores of tests in that cognitive domain (see Supplemental Table 2). All z-scores used are corrected for age and educational level. Correlations with *Dprime* and criterion included age, education, cognitive tasks, activities of daily living (Blessed Dementia Scale subscale), behavioural changes (Blessed Dementia Scale subscale) and total Blessed Dementia Scale. Although the cognitive battery we used did not yield a global cognitive index, we also computed a composite of the four composites as a proxy to the global MoCA score we used for healthy controls. The same correlations were also performed in the healthy control group between *Dprime*, criterion, MoCA, adjusted-MoCA (corrected for age and educational level), age and education.

As it could be argued that impairment in *Dprime* is secondary to impairment in other cognitive domains, we ran a multiple regression model for the MCI patients’ sample with relevant demographic measures (as determined by previous multiple regression for *Dprime*) and composite scores of all other cognitive measures as predictors of *Dprime*.

## Results

Demographic information, MoCA scores and adjusted-MoCA scores are shown in Table 1. There was a significant difference between groups for sex (*X(1)* = 5.4, p < 0.05) and age (*F*(1) = 4, *p* < 0.05) and a marginal significant difference for education (*F*(1) = 3.3, *p* = 0.07), with higher age and lower education in the MCI group. All the remaining models presented, therefore, included age, education and sex as predictors as well.

### Regression model for Dprime

**Figure 1.**
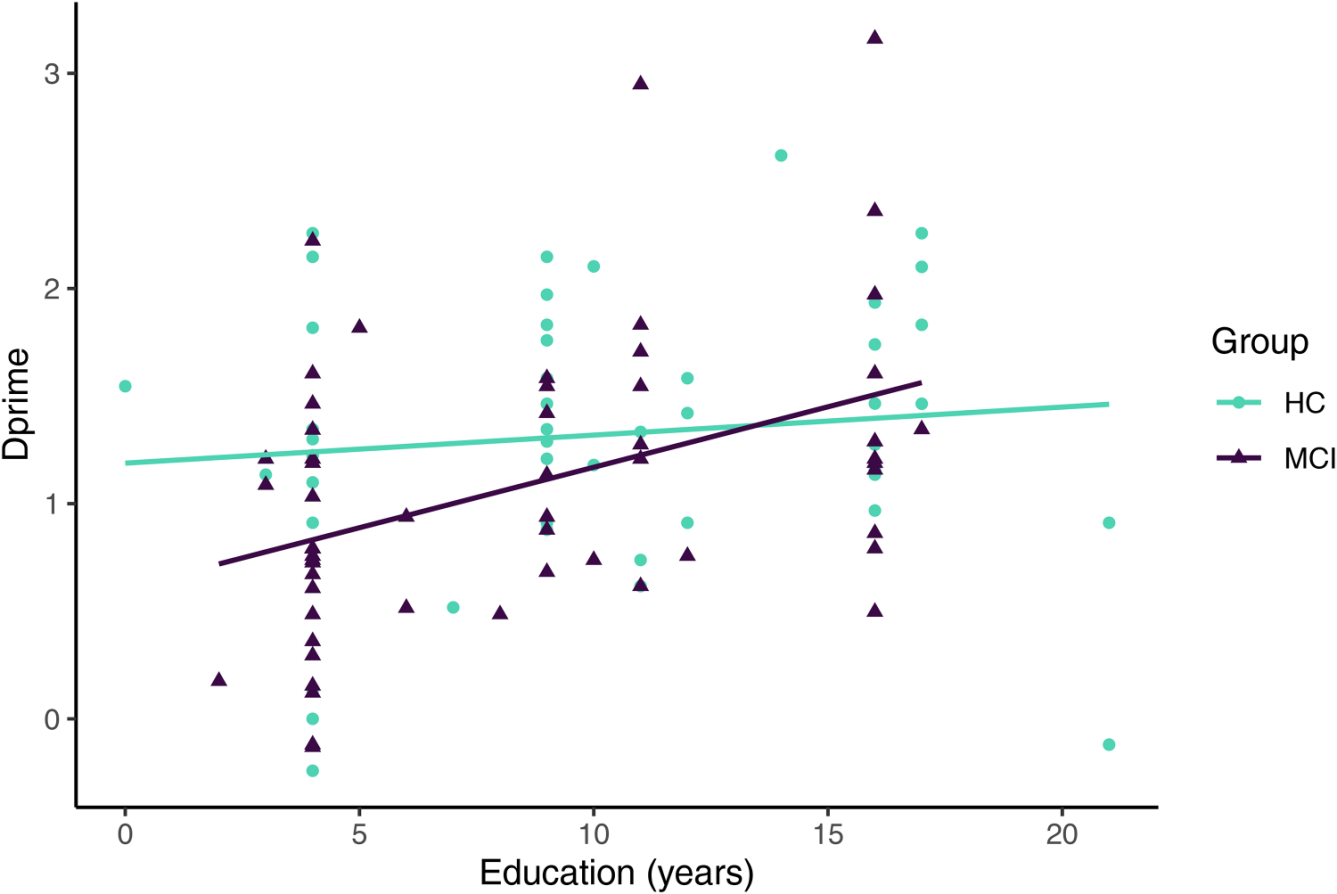
Discrimination performance in Dprime for MCI patients and healthy controls and educational level in years.

A multiple regression analysis was used to test if group, education, age and sex were significant predictors of *Dprime*. The overall regression model was significant (R^2^ = 0.16, R^2^-Adjusted = 0.12, F(5, 100) = 3.75, p = 0.01). Group was a significant predictor of *Dprime* (β = −0.54, t = −2.14, p = 0.04). Importantly, we found a marginal interaction between group and education (β = 0.05, t = 1.91, p = 0.06), in which increases in education decreased differences in *Dprime* between groups (Figure 1). Age, education and sex were not significant predictors of *Dprime* (age: β = −0.01, t = −0.95, p = 0.34; education: β = 0.01, t = 0.38, p = 0.71; sex: β = −0.18, t = −1.41, p = 0.16). We did not find influential observations as determined by cook's D (cook's D < 0.5).

As it could be argued that participants were not specifically assessing trustworthiness but other social traits as well, we also report two separate models for two other social traits: dominance and attractiveness. In both dominance model and attractiveness model, none of the previous predictors reached significance and the overall models were not significant (Supplemental Table 1).

### Regression model for criterion

We performed a multiple linear regression to test if group, age, education and sex were predictors of response bias. The overall model was not significant (R^2^ = 0.01, Adjusted R^2^ = − 0.04, F(4, 101) = 0.08, p = 0.99). None of the predictor variables reached significance (group: β = 0.01, t = 0.02, p = 0.93; education: β = 0.00, t = 0.14, p = 0.89; sex: β = 0.02, t = 0.19, p = 0.85; age: β = 0.00, t = 0.51, p = 0.61).

### Correlation with functionality and other cognitive measures in MCI patients

We found a significant positive correlation between *Dprime* and education (r (56)= 0.41, p = 0.001) and a significant positive correlation between *Dprime* and the visual composite (r (56) = 0.39, p = 0.003). We found a marginal positive correlation between Dprime and the attention composite (r(56) = 0.24, p = 0.07). Additionally, we found a positive weak but significant correlation between a global index of cognitive function and *Dprime* (r(56) = 0.28, p = 0.03).

**Figure 2.**
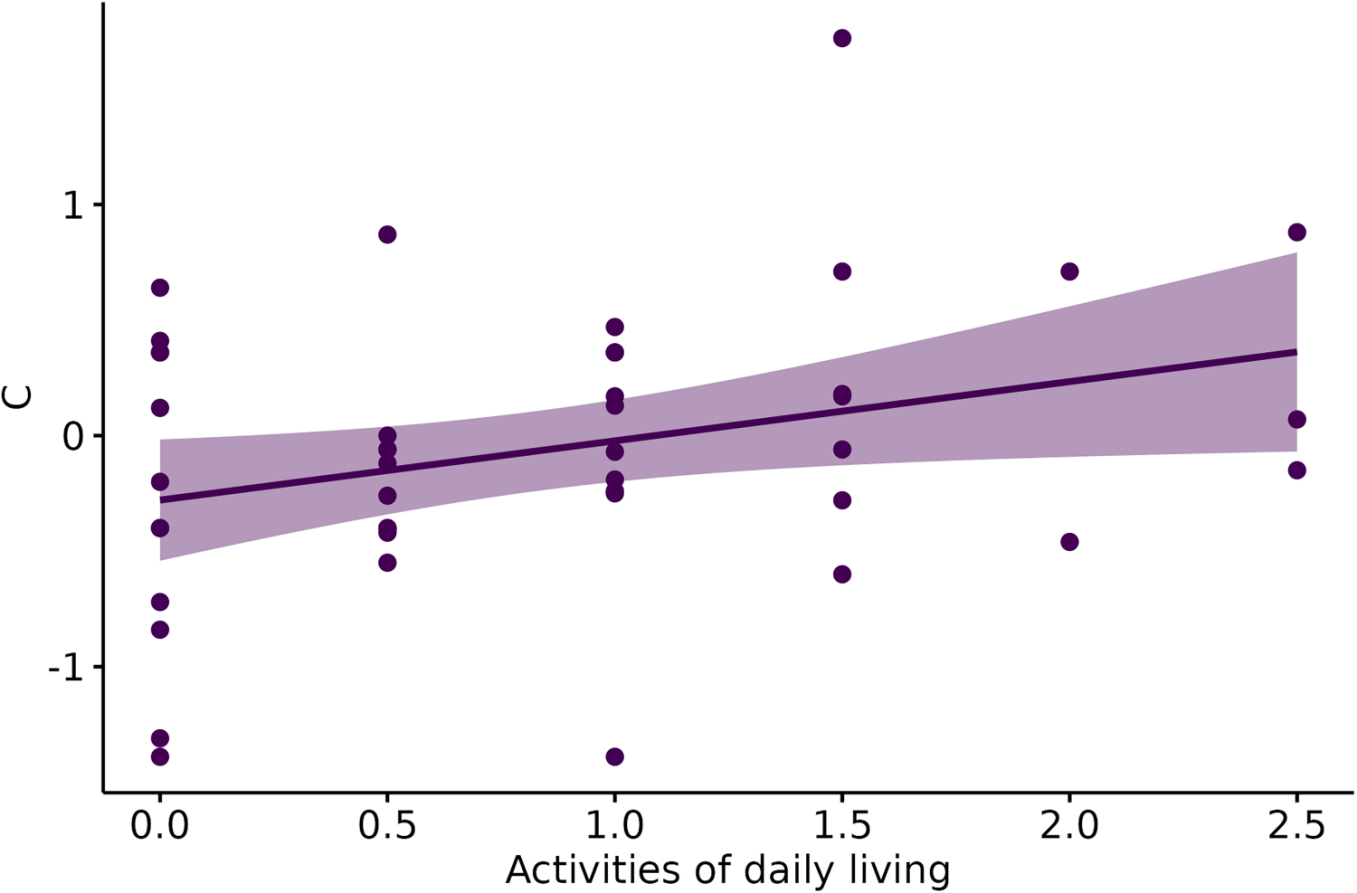
Correlation between criterion and impairment in activities of daily living. Shaded area represents confidence interval at 95%.

We also found a positive correlation between criterion and Activities of Daily Living (r (43) = 0.32, p = 0.03; Figure 2), wherein higher criterion (more conservative bias) was associated with higher impairment in activities of daily living. We did not find any significant correlation with other cognitive measures or with behavioral alterations.

### Correlation with functionality and other cognitive measures in healthy controls

We found a significant positive correlation between *Dprime* and MoCA (r (45) = 0.38, p = 0.01) and a marginal positive correlation between *Dprime* and adjusted-MoCA (r(45) = 0.24, p = 0.10). We also found a negative correlation between criterion and MoCA adjusted (r (45) = −0.36, p = 0.01), wherein higher criterion (more conservative bias) was associated with lower adjusted-MoCA scores.

### Regression model of dprime with other cognitive measures as predictors

A multiple regression analysis was used to test if education's effect on *Dprime* in the MCI group might be an indirect consequence of more severe cognitive impairments in patients with lower education. Educational level and cognitive composites were included as predictors of *Dprime*. The overall regression model was significant (R^2^ = 0.26, R^2^-Adjusted = 0.19, F(5, 52) = 3.68, p = 0.006). Education was the only significant predictor of *Dprime* (β = 0.04, t = 2.14, p = 0.037). Memory, attention, executive function and visual composite were not significant predictors of *Dprime* (memory composite: β = − 0.004, t = −0.56, p = 0.57; attention composite: β = 0.10, t = 1.36, p = 0.18; executive composite: β = −0.014, t = −1.19, p = 0.24; visual composite: = 0.08, t = 1.38, p = 0.17).

Similarly, we ran a multiple regression analysis to test if a lower global cognitive impairment in patients with lower education could explain the effect of education on *Dprime*. Educational level and global cognitive index were included as predictors of *Dprime*. Education remained as the only significant predictor of *Dprime* (β = 0.05, t = 2.73. p = 0.01) and global cognitive impairment did not reach significance (β = 0.17, t = 1.14. p = 0.26).

## Discussion

In the present study we aimed to investigate impairment in social trait inference in patients with early cognitive decline. We found that MCI patients already exhibited differences in the sensitivity to trustworthiness from faces compared to healthy controls. Interestingly, as what is described for other cognitive domains, we found that education qualified this difference by reducing it as years of education increased. The effect of group in sensitivity to trustworthiness could not be better explained by the inference of other social traits, specifically dominance and attractiveness, or by severity of cognitive impairment, or by impairment in other cognitive domains (for instance, executive functions). In patients, sensitivity to trustworthiness from faces was related to their visuoperceptive performance and to their global cognitive impairment. We did not find differences in response bias between groups and response bias did not differ from zero. However, patient's conservative response bias was correlated with more impairment in activities of daily living reported by a family member. Consistently, in healthy controls, more conservative response bias was related to less cognitive integrity.

On a 2-alternative forced-choice task, we found that increases in educational level decreased the difference in sensitivity to facial trustworthiness between MCI patients and healthy controls. This result, was irrespective of response bias, meaning that even though on average patients did not show a tendency to over-trust or distrust others, their ability to correctly perceive trustworthiness cues was impaired, as a function of education. The protective role of education on onset of cognitive symptoms and neurodegeneration has been extensively reported with higher educational level associated with later onset of cognitive symptoms,^37,49,50^ lower baseline neuropathology^51^ and more preserved brain metabolism despite the neuropathological burden.^52^ Our results align closely with this hypothesis, as in higher educational levels there were virtually no differences in performance between MCI patients and healthy controls, suggesting a protective role of education in sensitivity to trustworthiness. Importantly, however, the protective effect of education might weaken over disease progression, such that in advanced stages of AD pathology the cognitive advantage of education seems to be minimal.^37^ It is important to note that the protective role of education in the sensitivity to facial cues of trustworthiness was independent of its effect on other cognitive measures. The presence of altered sensitivity to this social trait in these patients might be relevant for their safety as trustworthiness perception cues approach/avoidance behavior^23,30^ and together with other cognitive impairments may increase vulnerability to being deceived.^18^ This result is in line with previous findings of impairment in facial emotion recognition and theory of mind in patients with MCI, suggesting a broader impairment in social cognitive function in these patients that may relate to early and subtle changes in social behavior.

We found that more impairment in activities of daily living was associated with a higher probability of perceiving others as untrustworthy. Contrary to sensitivity, which is usually considered as a perceptual marker, response bias reflects a more decisional aspect of trait inference, i.e., the amount of signal that needs to be present for the person to consider other as trustworthy. The impact of increased suspiciousness on others, independently of sensitivity ability, would most probably affect social and relational daily life. To our knowledge this is a novel result that connects a measure of disease severity to a tendency to perceive others in a more negative and suspicious way. This would be consistent with findings of early pathological deposition of tau-protein in the amygdala in the early stages of AD,^20^ a site known to be related to trustworthiness perception^19^ and trust-based decision.^53^ Additionally, in MCI patients more atrophy of the amygdala, orbitofrontal cortex and anterior cingulate was associated with higher levels of aggression.^54^ Lesion to the orbitofrontal cortex and anterior cingulate cortex have also been associated with disinhibition and abulia, respectively. This frontolimbic system seems to be common for both social cognitive function^19^ and the presence of behavioral alterations^55^ suggesting that an objective measurement of social cognitive impairment might overcome difficulties in subjective measurement of behavioral change by family members. The perception of others as untrustworthy, as disease progresses, could be a result of dysfunction of frontolimbic areas also related to the emergence of behavioral changes. Importantly, the combination of increased suspiciousness of others despite increased dependence on others found in these patients is of great importance for healthcare provision that relies on person-centered trust-building interventions.^56^ Additionally, this informs general medical practice by highlighting that MCI patient's perception of healthcare providers may hinder treatment adherence and negatively impact the patient-provider relationship.^17^

In healthy samples, lower trust has also been linked to lower grey matter volumes of the bilateral dorsomedial prefrontal cortex, left dorsolateral prefrontal cortex, left posterior cingulate cortex and bilateral precuneus. Both decreased grey matter volume in these areas and lower trust were also associated with higher depressive symptomatology^57^ suggesting that less trusting patients might be at risk of having more depressive symptoms. Complementary to this, higher positivity bias in the updating of undesirable situations and higher perceived trustworthiness of untrustworthy-looking faces was correlated with larger volume of the dorsal cingulate cortex and less activation of the amygdala, respectively. These results align with the presence of a positivity effect described in healthy aging.^32,33^ In the healthy control group, according to SST,^32,33^ we found that healthy controls with more liberal response bias had higher cognitive integrity. This result is not in line with predictions from the degradation hypothesis posed by the aging-brain model.^34^ Instead, our results provide evidence supporting the SST and extend it by suggesting that the positivity effect in social trait inference not only depends on unimpaired cognitive function, but also that it is greatest for more intact cognitive function.

In the patient group, the correlation between sensitivity to trustworthiness and visuoperceptive capacity was expected and seems to reflect the visuoperceptive nature of the task. According to the overgeneralization hypothesis,^11,12,58^ social trait inference derived from faces is based on the visual resemblance of the target face with the person's template of a trustworthy face (or positively charged emotional expression, see^30^), so impairment in visuoperceptive function is expected to disrupt this comparison therefore disrupting perceived trustworthiness. Nevertheless, it is interesting to note that, in this study, this correlation is weak which suggests that social trait inference may still occur in the presence of explicit visuoperceptive impairment. In accordance to this, the visual composite score was not a predictor of *Dprime.* Also, patients with acquired and developmental prosopagnosia, have shown no differences from controls in inferring social traits from faces even though they could not match them perceptually.^59,60^ Finally, in this group, we found that sensitivity scores had a weak but significant positive correlation with a global index of cognitive impairment which may reflect the interdependence between social and non-social cognitive function, supporting a shared trajectory in aging.

We found a positive correlation between sensitivity to trustworthiness and MoCA scores in healthy controls. Nevertheless, this relationship failed to reach significance when we used MoCA scores adjusted for age and education, suggesting that the initial correlation could be indirect, such that both sensitivity and unadjusted MoCA scores were positively correlated with education. Despite this, the marginal correlation between sensitivity and adjusted MoCA scores, is in line with our results from the MCI group, in which the global index of cognitive impairment was also positively correlated with sensitivity to trustworthiness. Once more, we believe this blurs the separation between social and non-social cognitive functions in clinical practice and research and it also questions previous conceptualizations of social cognition as a result of an impairment in others cognitive domains.

In conclusion, this study shows original evidence of an education-dependent impairment in trustworthiness perception in early cognitive decline. We show novel evidence for the protective role of education in a social cognitive function, suggesting a shared trajectory with ‘standard’ cognitive functions. We extend findings on the determinants of the positivity effect described in healthy aging wherein higher cognitive integrity maximizes it. These findings contribute to bridging evidence with research on emotion processing and theory of mind into a comprehensive body of knowledge in social neuroscience, informing patient care interventions for both family and healthcare providers and signaling the importance of considering education as a buffer for social trait inference impairment in clinical neuropsychological assessment.

## Supplemental Material

sj-docx-1-alz-10.1177_13872877261426496 - Supplemental material for Perception of facial trustworthiness in mild cognitive impairment Supplemental material, sj-docx-1-alz-10.1177_13872877261426496 for Perception of facial trustworthiness in mild cognitive impairment by Marta Granadeiro, Leonel Garcia-Marques, Marco Torrado and Isabel Pavão Martins in Journal of Alzheimer's Disease
