# Supplementary material for: Perception of facial trustworthiness in mild cognitive impairment
Source: J Alzheimers Dis. 2026 Mar 13;110(4):1667–77. doi: 10.1177/13872877261426496 (PMC13058154; doi:10.1177/13872877261426496)
Supplement: sj-docx-1-alz-10.1177_13872877261426496 - Supplemental material for Perception of facial trustworthiness in mild cognitive impairment [file sj-docx-1-alz-10.1177_13872877261426496.docx]

**Supplemental Material**

**Perception of facial trustworthiness in mild cognitive impairment**

**Supplemental Table 1.** Regression results (standardized β, t, p-value for the coefficients, R^2^ Adjusted, F, p-value for the overall model) for group, age, education and sex as predictors of sensitivity to dominance and attractiveness as measured by dominance *Dprime* and attractiveness *Dprime*, respectively.

| Predictors | β | t | p | R^2^ | F(5, 100) | p |
| --- | --- | --- | --- | --- | --- | --- |
| Dominance Model |  |  |  | - 0.01 | 0.77 | 0.57 |
| Group | - 0.04 | - 0.22 | 0.83 |  |  |  |
| Age | 0.01 | 1.11 | 0.27 |  |  |  |
| Education | - 0.01 | - 0.84 | 0.40 |  |  |  |
| Sex | 0.02 | 0.29 | 0.78 |  |  |  |
| Attractiveness Model |  |  |  | 0.04 | 1.87 | 0.11 |
| Group | - 0.24 | - 1.28 | 0.21 |  |  |  |
| Age | 0.00 | 0.43 | 0.67 |  |  |  |
| Education | 0.01 | 0.64 | 0.53 |  |  |  |
| Sex | - 0.08 | - 0.80 | 0.43 |  |  |  |

**Supplemental Table 2.** Mean scores and standard deviation of neuropsychological assessment tests

|  | MCI | |
| --- | --- | --- |
|  | M | SD |
| **Attention** |  |  |
| Letter Cancellation | 0.04 | 1.35 |
| Digit Span Forward | 0.34 | 1.76 |
| Trail Making Test A | -0.58 | 1.21 |
| **Memory** |  |  |
| Logical Memory | -1.66 | 1.29 |
| Word-Pair Associates | -1.30 | 0.95 |
| California Verbal Learning Test (learning sum 1-5) | -3.17 | 1.59 |
| **Executive function** |  |  |
| Digit Span Backwards | -0.15 | 0.99 |
| Stroop Interference | -1.07 | 0.90 |
| Trail Making Test B-A | -1.1 | 0.78 |
| **Visual** |  |  |
| Cube Copy | 0.31 | 1.30 |
| Incomplete Letters (VOSP) | -1.42 | 3.64 |
| Number Location (VOSP) | 0.97 | 1.22 |

M: mean of z-scores; SD: standard deviation of z-scores

**Supplemental Procedure**

*Brief description of tests*

**Letter Cancellation** - The patient is presented with a 10x10 array of repeated letters. They are asked to cross every letter A they can find as fast as possible. The score is the number of correctly crossed “A”s divided by time.

**Digit Span forward** - a sequence of digits is read to the patient, who is asked to repeat them in the same order. Sequences start with 3 numbers and length increases as patients correctly repeat the sequence. The score is the longest sequence of digits the patient was able to correctly repeat.

**Trail Making Test A** - The patient is presented with an array of 25 numbers, inside circles distributed randomly in the page. They are instructed to connect all the numbers with a line in the correct order as fast as they can. They start with an example of 8 numbers on one page and, if they do not need further clarification and perform the example correctly, they are asked to repeat the same with 25 numbers. The score is the total time the patient takes to correctly connect all the numbers. The task is interrupted after 2.5 minutes.

**Logical Memory** - The patient is read a short paragraph with a story. Afterwards, they are asked to repeat all the details they can remember from the story from memory. Then, they are read a second story and asked to do the same. Final score is the mean between the number of details the patient correctly recalled for each story.

**Word-Pair associates** - The patient is read a set of 10 pairs of words. After this, they are asked to recall which target word was paired with which prompt word. This is repeated 2 times. The score is the number of correctly recalled pairs of words, score in easier associate pairs is worth half of the score in more difficult pairs.

**California Verbal Learning Test** - the patient is read a list of 15 unrelated words. After this, they are asked to recall as many words from the list as possible. This is repeated across 5 trials. The score is the sum of words they recalled in all the trials.

**Digit Span backwards** - a sequence of digits is read to the patient, who is asked to repeat them in reverse order. Sequences start with 2 numbers and length increases as patients correctly repeat the sequence. The score is the longest sequence of digits the patient was able to correctly repeat in reverse order.

**Stroop Test** - In the first trial, the patient is presented with columns with the words “Red”, “Green” and “Blue”. The patient is asked to read out loud each word as fast as they can. In the second trial, the patient is presented with columns with “xxxx” written in different colors and is asked to name all the colors presented as fast as they can. Finally, the patient is presented with columns with the words “Red”, “Green” and “Blue” written in different colors. They are instructed to name the colors they see, ignoring the meaning of the words. For instance, if the word “Green” was written in blue color, they should answer “blue”. The score is the number of correctly named colors of the last trial, proportionally discounting on the number of correctly read words of the first trial and number of correctly named colors of the second trial (final score = third trial - (first trial*second trial/(first trial + second trial)).

**Trail Making Test B** - The patient is presented with an array of numbers and letters inside circles distributed randomly in the page. They are instructed to connect, in alternating order, numbers and letters. They start with an example of 4 numbers and 4 letters on one page and, if they do not need further clarification and perform the example correctly, they are asked to repeat the same with all the numbers and letters. Score is the total time the patient takes to correctly connect all the numbers. The task is interrupted after 5 minutes.

**Cube Copy** - the patient is presented with a drawing of a cube and is asked to copy it as accurately as they can. The score is unrecognizable drawing (0 points), correct gross copy without perspective (1 point), correct copy with perspective but not totally correct (2 points), completely correct copy (3 points).

**Incomplete Letters (VOSP)** - The patient is sequentially presented with 20 fragmented uppercase letters. They are asked to name each of the fragmented letters. The score is the number of letters correctly named.

**Number Location (VOSP)** - The patient is presented, in each trial, with two squares (one above the other). The upper square has numbers distributed inside. The lower square has a black dot, which is in the same relative position as one of the numbers of the upper square. The patient is asked to find the number which is in the corresponding position of the dot.

*Social trait inference task*

​
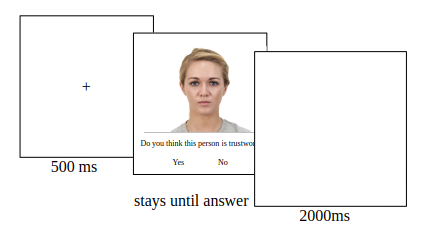
​

​​

**Supplemental Figure 1.** Social trait inference task.

**Supplemental Table 3.** Normative data from the Chicago Face Database used to classify each face in dominance and attractiveness.

|  | Chicago Face Database – normative data | | | | | | Defined classification | | | |
| --- | --- | --- | --- | --- | --- | --- | --- | --- | --- | --- |
| Stimuli | Trust. | Mean | Dom. | Mean | Attract. | Mean | Trustworthiness | Dominance | Attractiveness |  |
| Stim 1 | 2.86 | 3.43 | 3.90 | 2.83 | 2.14 | 3.23 | low | high | low |  |
| Stim 2 | 4.25 | 3.43 | 2.32 | 2.83 | 4.68 | 3.23 | high | low | high |  |
| Stim 3 | 4.27 | 3.43 | 2.15 | 2.83 | 4.19 | 3.23 | high | low | high |  |
| Stim 4 | 4.28 | 3.43 | 2.28 | 2.83 | 4.41 | 3.23 | high | low | high |  |
| Stim 5 | 4.50 | 3.43 | 2.38 | 2.83 | 4.67 | 3.23 | high | low | high |  |
| Stim 6 | 4.46 | 3.43 | 1.93 | 2.83 | 4.21 | 3.23 | high | low | high |  |
| Stim 7 | 4.48 | 3.43 | 2.43 | 2.83 | 3.81 | 3.23 | high | low | high |  |
| Stim 8 | 4.31 | 3.43 | 2.07 | 2.83 | 3.08 | 3.23 | high | low | low |  |
| Stim 9 | 2.30 | 3.43 | 4.13 | 2.83 | 2.52 | 3.23 | low | high | low |  |
| Stim 10 | 4.15 | 3.43 | 3.58 | 2.83 | 3.54 | 3.23 | high | high | high |  |
| Stim 11 | 4.16 | 3.43 | 2.04 | 2.83 | 2.40 | 3.23 | high | low | low |  |
| Stim 12 | 2.54 | 3.43 | 2.83 | 2.83 | 2.54 | 3.23 | low | high | low |  |
| Stim 13 | 2.75 | 3.43 | 3.89 | 2.83 | 1.89 | 3.23 | low | high | low |  |
| Stim 14 | 2.38 | 3.43 | 3.76 | 2.83 | 1.55 | 3.23 | low | high | low |  |
| Stim 15 | 4.33 | 3.43 | 2.92 | 2.83 | 4.60 | 3.23 | high | high | high |  |
| Stim 16 | 2.74 | 3.43 | 2.63 | 2.83 | 2.48 | 3.23 | low | low | low |  |
| Stim 17 | 4.28 | 3.43 | 3.20 | 2.83 | 5.08 | 3.23 | high | high | high |  |
| Stim 18 | 2.45 | 3.43 | 4.00 | 2.83 | 2.03 | 3.23 | low | high | low |  |
| Stim 19 | 2.63 | 3.43 | 4.88 | 2.83 | 2.11 | 3.23 | low | high | low |  |
| Stim 20 | 4.63 | 3.43 | 2.70 | 2.83 | 4.19 | 3.23 | high | low | high |  |
| Stim 21 | 4.17 | 3.43 | 3.71 | 2.83 | 4.85 | 3.23 | high | high | high |  |
| Stim 22 | 4.14 | 3.43 | 2.75 | 2.83 | 2.86 | 3.23 | high | low | low |  |
| Stim 23 | 2.41 | 3.43 | 2.19 | 2.83 | 2.11 | 3.23 | low | low | low |  |
| Stim 24 | 4.21 | 3.43 | 4.14 | 2.83 | 3.61 | 3.23 | high | high | high |  |
| Stim 25 | 4.50 | 3.43 | 2.92 | 2.83 | 4.56 | 3.23 | high | high | high |  |
| Stim 26 | 2.67 | 3.43 | 3.23 | 2.83 | 2.76 | 3.23 | low | high | low |  |
| Stim 27 | 4.31 | 3.43 | 2.21 | 2.83 | 5.24 | 3.23 | high | low | high |  |
| Stim 28 | 2.70 | 3.43 | 2.89 | 2.83 | 3.44 | 3.23 | low | high | high |  |
| Stim 29 | 4.57 | 3.43 | 3.50 | 2.83 | 3.48 | 3.23 | high | high | high |  |
| Stim 30 | 2.48 | 3.43 | 4.36 | 2.83 | 2.78 | 3.23 | low | high | low |  |
| Stim 31 | 2.45 | 3.43 | 3.09 | 2.83 | 4.30 | 3.23 | low | high | high |  |
| Stim 32 | 2.32 | 3.43 | 4.22 | 2.83 | 3.50 | 3.23 | low | high | high |  |
| Stim 33 | 4.04 | 3.43 | 2.50 | 2.83 | 4.46 | 3.23 | high | low | high |  |
| Stim 34 | 2.37 | 3.43 | 4.38 | 2.83 | 3.27 | 3.23 | low | high | high |  |
| Stim 35 | 4.52 | 3.43 | 2.22 | 2.83 | 3.73 | 3.23 | high | low | high |  |
| Stim 36 | 2.65 | 3.43 | 3.69 | 2.83 | 1.88 | 3.23 | low | high | low |  |
| Stim 37 | 2.43 | 3.43 | 2.69 | 2.83 | 2.21 | 3.23 | low | low | low |  |
| Stim 38 | 2.46 | 3.43 | 2.80 | 2.83 | 2.28 | 3.23 | low | low | low |  |
| Stim 39 | 2.48 | 3.43 | 4.62 | 2.83 | 2.52 | 3.23 | low | high | low |  |
| Stim 40 | 2.31 | 3.43 | 3.33 | 2.83 | 2.41 | 3.23 | low | high | low |  |

Mean refers to the general mean of all faces in the database for each social trait (which includes 597 faces). Trust. = mean trustworthiness rating for each face stimulus. Dom. = mean dominance rating for each face stimulus. Attract. = mean attractiveness rating for each face stimulus.
